# Inositol metabolism as a broad-spectrum antiviral target

**DOI:** 10.3389/fmicb.2025.1620775

**Published:** 2025-08-26

**Authors:** Kunlakanya Jitobaom, Prasert Auewarakul

**Affiliations:** ^1^Department of Microbiology, Faculty of Medicine Siriraj Hospital, Mahidol University, Bangkok, Thailand; ^2^Emerging Infectious Research Unit, Research Department, Faculty of Medicine Siriraj Hospital, Mahidol University, Bangkok, Thailand

**Keywords:** phosphatidylinositol, inositol monophosphatase, phosphatidylinositol kinase, myo-inositol metabolism, broad-spectrum antiviral, host-virus interaction

## Abstract

Inositol plays many important roles in cellular processes through its various derivatives including phosphatidylinositol phosphates. Viruses use phosphatidylinositol phosphates for their replication in multiple processes including entry, formation of replication organelles, assembly and release. For these processes, viruses recruit phosphatidylinositol kinases to meet their demand of phosphatidylinositol phosphates. Inhibitors of phosphatidylinositol kinases have been shown to inhibit various viruses. The complexity of various types and isoforms of phosphatidylinositol kinases can be a problem in developing a broad-spectrum antiviral as different viruses use various types and isoforms of the enzyme. Inositol monophosphatase is an enzyme required for both *de novo* biosynthesis and intracellular recycling of inositol. It can provide a chokepoint to limit the availability of cellular inositol, phosphatidylinositol, and phosphatidylinositol phosphates. It can be a promising target for broad-spectrum antiviral development.

## 1 Introduction

Inositol, hexahydroxy-cyclohexane, is a cyclic polyol which composed of a six-carbon ring, and each carbon is hydroxylated. There are nine stereoisomers of inositol (cis-, epi-, allo, myo-, neo-, scyllo-, L- chiro-, D- chiro-, and muco-inositol). Seven isomers are found in nature, except for epi- and allo-inositol, while myo-inositol (MI) is the most abundant form (Loewus and Loewus, 1983; Thomas et al., 2016).

Inositol is found in both prokaryotes and eukaryotes. The primary role of inositol in prokaryotes is to regulate physiological osmolarity and cellular pH (Hazra and Nandy, 2016). Inositol is also essential for osmoregulation to protect the cells from hyperosmolarity in the mammalian brain and kidney cells (Garcia-Perez and Burg, 1990; Dai et al., 2016). However, more diverse functions were observed in eukaryotes. Inositol serves as a precursor to several derived metabolites, including inositol phosphates (InsP), phosphatidylinositol (PtdIns), various forms of phosphorylated PtdIns or phosphoinositides (PPIns), and inositol pyrophosphates (PP-InsPs).

PtdIns is a ubiquitous phospholipid found in the cytoplasmic leaflet of the plasma membrane, membrane-bound organelles, and endoplasmic reticulum (ER), where it is synthesized. It is also a precursor of PPIns, which act as second messengers in multiple signaling pathways and are involved in diverse biological processes, including actin cytoskeletal organization, membrane dynamics, and lipid metabolism and transport. Moreover, the network of phosphorylated inositol derivatives coordinates the cellular response to nutrients and the balance between energy production and utilization on two primary metabolic pathways: AMP-activated protein kinases (AMPK) and the mammalian target of rapamycin (mTOR) (Tu-Sekine and Kim, 2022). PPIns also participate in immune cell functions, cellular stress response, apoptosis, and secretion (Chen et al., 1998; De Craene et al., 2017; Chhetri, 2019; Bizzarri et al., 2023). PPIns levels are regulated by several phosphatidylinositol kinases (PIKs) and phosphatases, which are differently distributed across various subcellular compartments. This distribution results in the different localization of PPIns and their roles in several metabolic pathways (Beziau et al., 2020).

Viruses exploit host cellular pathways to enhance their replication (Fischl and Bartenschlager, 2011). Common targets manipulated by viruses include lipid metabolism and transport, as well as lipid-mediated signal transductions. Alterations in lipid metabolism and transport are required to redirect cellular lipids to favor viral replication and assembly. Additionally, viruses evade the host immune response by interfering with signal transduction mechanisms. Most viruses suppress interferon (IFN)-induced transcriptional responses, including the Janus kinase (JAK)/signal transducers and activators of transcription (STAT) signaling pathways (Alcami and Koszinowski, 2000). For instance, the NS4B protein of the dengue virus (DENV) blocks IFN signaling by reducing the nuclear translocation of STAT1, thereby affecting the JAK/STAT pathway (Ezeonwumelu et al., 2021). The viral strategy for manipulating host cellular lipids or disrupting signal transductions can be mediated through inositol metabolism by targeting host PIKs, phosphatases, and their accessory proteins. For instance, viruses recruit phosphatidylinositol 4-kinases (PI4Ks) to establish phosphatidylinositol 4-phosphate (PI4P)-enriched replication organelles (ROs) to concentrate the lipids required for their replication within ROs (Berger et al., 2009; Berger et al., 2011; McPhail and Burke, 2023).

Therefore, a comprehensive understanding of the importance of inositol, PtdIns, and PPIns metabolisms, especially the roles of PIKs involved in viral replication cycles can provide a novel approach for developing antiviral strategies.

## 2 Biosynthesis of myo-inositol and its derivatives

Organisms mainly rely on the cellular biosynthesis of MI, either the *de novo* synthesis from glucose or the catabolism of PtdIns, PPIn, and InsP (Gonzalez-Uarquin et al., 2020). However, inositol could also be acquired from food consumption. The intracellular inositol can be imported via inositol transporters (Gonzalez-Salgado et al., 2012). Inositol transporters, conserved across bacteria to animals, mediate uptake and regulate intracellular distribution. There are two groups of inositol transporters: sodium ion-coupled and proton-coupled, which are located in the plasma membrane (Schneider, 2015). SMIT1 and SMIT2, sodium/myo-inositol transporters encoded by *SLC5A3* and *SLC5A11* (Hitomi and Tsukagoshi, 1994; Berry et al., 1995), share 43% amino acid sequence identity (Coady et al., 2002). SMIT1 mainly contributes to osmoregulation by controlling inositol accumulation in the cells of the brain and kidney. Upregulation of *SLC5A3* was observed in hypertonic and high osmolarity conditions (Yamauchi et al., 1995). The preferred substrate of SMIT1 and SMIT2 is inositol, with a Km value of 55 μM and 120 μM, respectively (Coady et al., 2002). Both SMIT1 and SMIT2 show low affinity to glucose (Hager et al., 1995; Coady et al., 2002). The mammalian proton-coupled inositol transporters (HMIT1) encoded by the *SLC2A13* gene are highly expressed in the brain, and the upregulation results in hypertonic conditions (Uldry et al., 2001). The affinity for myo-inositol shows a Km value of 100 μM. HMIT1 also binds with scyllo-, chiro-, and muco-inositol (Uldry et al., 2001).

**FIGURE 1:**
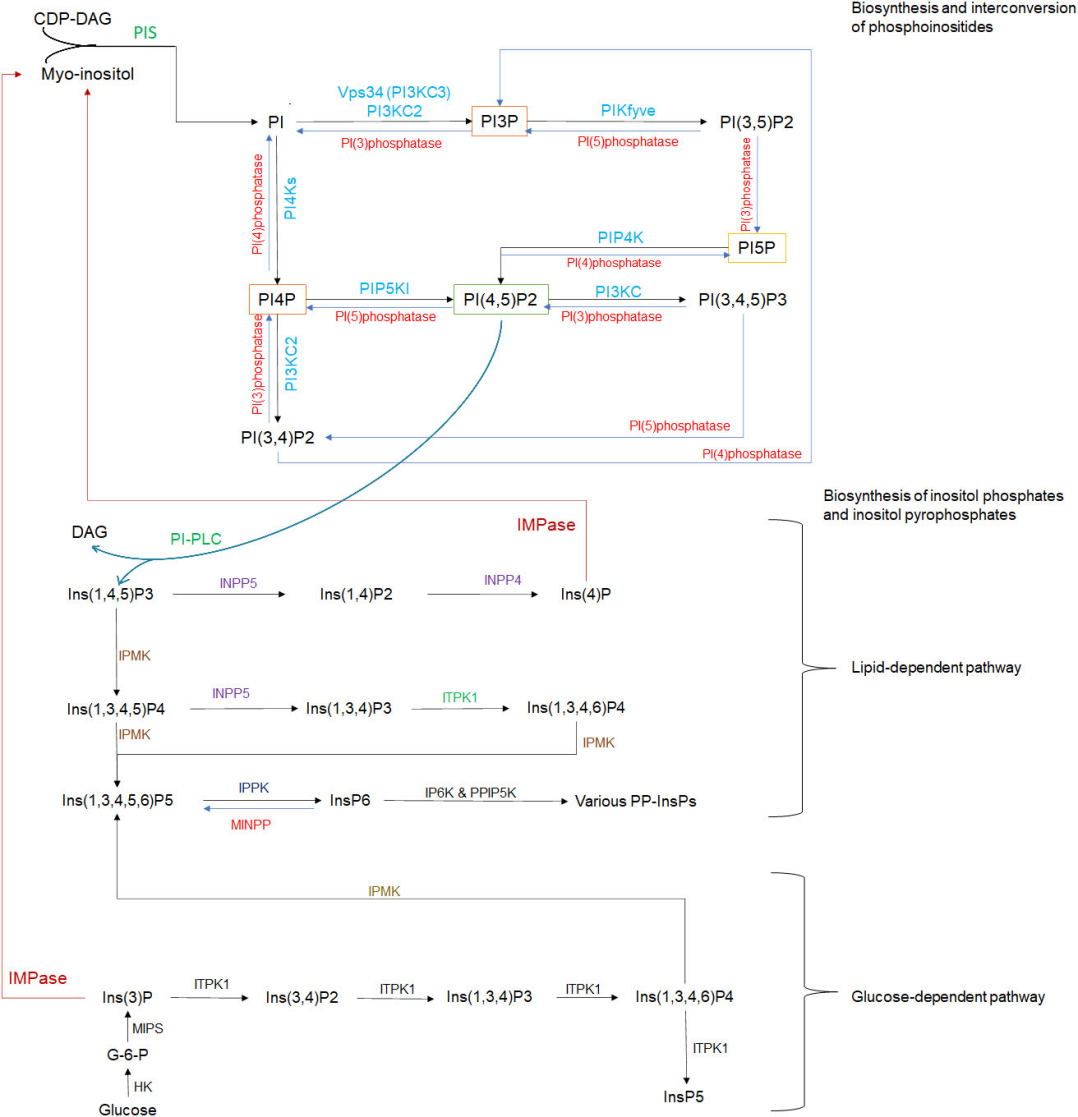
Biosynthesis and interconversion of phosphoinositides (PPIns), inositol phosphates (InsPs), and inositol pyrophosphates (PP-InsPs), mediated by various PIKs and phosphatases. The interconversion of seven PPIns is shown in the top panel. PIKs are represented in blue, while phosphatidylinositol phosphatases (PI phosphatases) are represented in red. Biosynthesis of InsPs and PP-InsPs is shown in the middle and bottom panels, respectively. CDP-DAG, cytidine diphosphate diacylglycerol; PI, phosphatidylinositol; PIS, phosphatidylinositol synthase; IMPase, inositol monophosphatase; DAG, diacylglycerol; PI-PLC, phosphatidylinositol phospholipase-C; INPP5, inositol polyphosphate-5-phosphatase; INPP4, inositol polyphosphate-4-phosphatase; IPMK, inositol polyphosphate multikinase; IPPK, inositol-pentakisphosphate 2-kinase; IP6K, inositol hexakisphosphate kinase; PPIP5K, diphosphoinositol-pentakisphosphate kinase; ITPK1, inositol-tetrakisphosphate 1-kinase; MINPP, multiple inositol polyphosphate phosphatase; HK, hexokinase; MIPS, myo-inositol 1-phosphate synthase.

**FIGURE 2:**
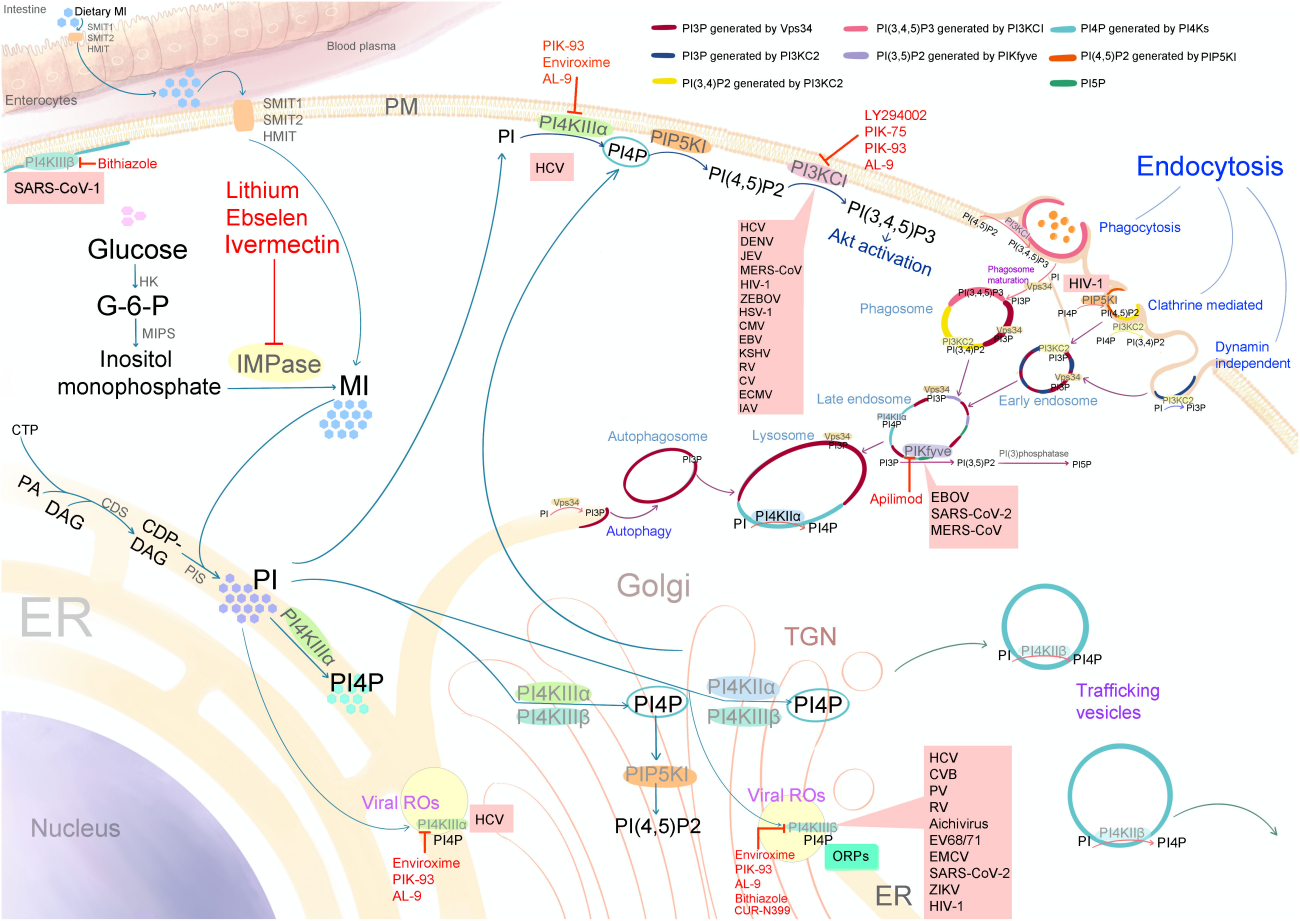
Schematic diagram illustrating the biosynthesis of myo-inositol (MI), phosphatidylinositol (PI), and various phosphoinositides (PPIns), along with the subcellular distribution of phosphatidylinositol kinases (PIKs), PPIns, viral target sites, and the targets of PIK inhibitors with antiviral activity (indicated by red letters and lines). SMIT1 and 2: sodium/myo-inositol transporters 1 and 2; HMIT, proton-coupled inositol transporter; PM, plasma membrane; ER, endoplasmic reticulum; TGN, trans-Golgi network; HK, hexokinase; MIPS, myo-inositol 1-phosphate synthase; G-6-P, glucose-6-phosphate; IMPase, inositol monophosphatase; CTP, cytidine triphosphate; PA, phosphatidic acid; DAG, diacylglycerol; CDP-DAG, cytidine diphosphate diacylglycerol; CDS, CDP-diacylglycerol synthase; PIS, phosphatidylinositol synthase; ROs, viral replication organelles; PI4KII (α and β), phosphatidylinositol 4-kinases type II; PI4KIII (α and β), phosphatidylinositol 4-kinases type III; PIKfyve, phosphatidylinositol 5-kinase; PI3KCI, phosphatidylinositol 3-kinase type I; PI3KC2, phosphatidylinositol 3-kinase type II; Vps34 or PI3KC3, vacuolar protein sorting 34 or phosphatidylinositol 3-kinase type III; PIP5KI, phosphatidylinositol-4-phosphate-5-kinase type I; ORPs, oxysterol-binding protein (OSBP)-related proteins; PI(3)phosphatase, phosphatidylinositol phosphatases. SARS-CoV (1 and 2), severe acute respiratory syndrome coronavirus; MERS-CoV, Middle East respiratory syndrome coronavirus; HCV, hepatitis C virus; DENV, dengue virus; ZIKV, Zika virus; JEV, Japanese encephalitis virus; CV, coxsackieviruses; CVB, coxsackievirus B; PV, poliovirus; RV, rhinovirus; EV68/71, enterovirus 68 and 71; EMCV, encephalomyocarditis virus; IAV, influenza A virus; EBOV, Ebola virus; ZEBOV, Zaire ebolavirus; HIV-1, human immunodeficiency virus-1; HSV-1, herpes simplex virus-1; CMV, cytomegalovirus; EBV, Epstein–Barr virus; and KSHV, Kaposi sarcoma–associated herpesvirus.

Inositol can be generated *de novo* from glucose-6-phosphate (G6P) to inositol-3-phosphate [Ins(3)P] by myo-inositol 1-phosphate synthase (MIPS, *ISYNA1*) (Eisenberg, 1967). Then the phosphate moiety is removed by inositol monophosphatase (IMPase, *IMPA1*, *IMPA2*) into free MI (Figures 1, 2) (Loewus et al., 1980; Bizzarri et al., 2023). Both MIPS and IMPase contribute significantly to maintaining free MI for biosynthesis of various derivatives. Additionally, MI can activate p53, which results in the upregulation of *ISYNA1* expression as positive feedback for MI generation (Bizzarri et al., 2023).

MI derivatives are classified into two main categories: lipid-associated and soluble forms. Lipid-associated MI derivatives include PtdIns and phosphorylated forms (phosphoinositides/PPIns), while soluble derivatives comprise InsP and PP-InsPs.

### 2.1 Biosynthesis of phosphatidylinositol and phosphoinositides

The synthesis of PtdIns occurs in the endoplasmic reticulum (ER) through the condensation of free MI and a liponucleotide, cytidine diphosphate diacylglycerol (CDP-DAG), facilitated by CDP-diacylglycerol–inositol 3-phosphatidyltransferase (C*DIPT* or phosphatidylinositol synthase; PIS) (Loewus and Loewus, 1983; Eisenberg and Parthasarathy, 1987). Meanwhile, CDP-DAG is synthesized in the ER from phosphatidic acid and cytidine triphosphate via CDP-diacylglycerol synthase (CDS) activity (Blunsom and Cockcroft, 2020). PtdIns predominantly localize to cellular membranes, including the nucleus, ER, Golgi complex, endosomes, and lysosomes (De Craene et al., 2017), and account for 10–20% of total cellular phospholipids in eukaryotic cells, whereas PPIns comprise only ∼1% (Di Paolo and De Camilli, 2006; Balla, 2013; see Figures 1, 2). PtdIns can be phosphorylated by different PIKs on the hydroxyl groups at positions three, four, and five. There are three main types of PPIns, which contain one, two, or three phosphate groups. The seven members of PPIns include phosphatidylinositol 3-phosphate (PI3P), phosphatidylinositol 4-phosphate (PI4P), phosphatidylinositol 5-phosphate (PI5P), phosphatidylinositol 3,4-bisphosphate [PI(3,4)P2], phosphatidylinositol 3,5-bisphosphate [PI(3,5)P2], phosphatidylinositol 4,5-bisphosphate [PI(4,5)P2], and phosphatidylinositol 3,4,5-trisphosphate [PI(3,4,5)P3].

The interconversion of the seven PPIns is mediated by several families of PIKs and phosphatidylinositol phosphatases, whose variant isoforms are localized in different subcellular compartments, resulting in varied distribution of PPIns (Figure 1). This determines their specific roles in different biological processes.

#### 2.1.1 Phosphatidylinositol 3-kinases (PI3Ks)

PI3Ks, membrane-associated PIKs, phosphorylate the inositol ring at the D3 position and comprise three subunits: p85 regulatory subunit, p55 regulatory subunit, and p110 catalytic subunit. PI3Ks were classified to three types, including type I, II, and III based on different structures and specific substrates (Katso et al., 2001).

PI3K type I (PI3KCI) mainly generates PI(3,4,5)P3 from PI(4,5)P2, which is a second messenger in PI3K/Akt/mTOR pathway (Amzel et al., 2008). PI3K type I can be further divided into type IA and type IB. Type IA PI3Ks are heterodimers of a regulatory subunit p85 and a catalytic subunit p110. The catalytic subunit p110 can be p110α, p110β, or p110δ isoforms, and only p110γ catalytic subunit is found PI3K type IB (Engelman et al., 2006). Based on different p110 catalytic subunits, PI3Ks can also be classified into four isoforms, including α, β, δ, and γ isoforms. PI3Ks are activated differently downstream of receptor tyrosine kinases (RTKs), G protein-coupled receptors and Ras GTPases (Yuan and Cantley, 2008; Vanhaesebroeck et al., 2010). Upon PI3K/Akt/mTOR pathway activation, PI3KCI produces PI(3,4,5)P3, which recruits Akt to the membrane by acting as the binding site. Akt is then phosphorylated by phosphoinositide-dependent protein kinase-1 (PDK1), activating mTOR signaling that regulates cell proliferation, survival, and motility (Paplomata and O’Regan, 2014).

PI(3,4,5)P3 is dephosphorylated at the D3 position by phosphatase and tensin homolog (PTEN), a tumor suppressor that function as PIP3-phosphatase, converting it back to PI(4,5)P2.

This results in the inactivation of the PI3K/Akt/mTOR pathway (Csolle et al., 2020). Mutation of PI3Ks have been implicated in various human cancers. PI3KCI inhibition has become a target in cancer therapeutics, as PI3K inhibitors can reduce cellular proliferation and promote cell death (Yang et al., 2019).

PI3K type II (PI3KC2) generates PI3P and PI(3,4)P2 from PI and PI4P, respectively, by phosphorylation at D3 position of the MI ring. PI3KC2 is resistance against wortmannin and LY294002 (Wang Y. et al., 2006). There are three isoforms, including PI3KC2α, PI3KC2β, and PI3KC2γ. PI(3,4)P2 produced by PI3KC2α promotes the maturation of clathrin-coated pits (CCPs) and facilitates membrane neck scission during clathrin-coated vesicle formation (Schöneberg et al., 2017). Moreover, the endocytic pools of PI3P produced by PI3KC2α mediate endosomal signaling, including that of RhoA, Rac1, and Rap1 (Yoshioka, 2021). PI3KC2β is also essential for the formation and maturation of CCPs through its recruitment via interaction with intersectin-1 (ITSN1), which stimulates actin filament formation at CCPs (Russo and O’Bryan, 2012; Almeida-Souza et al., 2018). PI3KC2γ mediates insulin-dependent production of endosomal PI(3,4)P2 that can extend the activation of endosomal Akt2 (Braccini et al., 2015).

PI3K type III (PI3KC3) is involved in intracellular membrane trafficking and autophagy. Its primary isoform, vacuolar protein sorting 34 (Vps34), phosphorylates PtdIns to generate PI3P, which is essential for the formation of early endosomes and autophagosomes (Li et al., 2024). Vps34 functions by forming two different complexes, which regulate the activity of different pathways. Complex I is involved in autophagy, whereas Complex II participates in endocytosis and membrane trafficking for vacuolar protein sorting (Backer, 2008; Jean and Kiger, 2014).

#### 2.1.2 Phosphatidylinositol 4-kinases (PI4Ks)

PI4Ks phosphorylate the D4 hydroxyl of the myo-inositol ring in PtdIns, producing PI4P. The produced PI4P then serves as a precursor for the synthesis of other PPIns. Moreover, PI4P can be exchanged for lipids being transported to the plasma membrane or organelle membranes. PI4Ks consist of four isoforms, which can be classified into two types based on domain organization and biochemical properties: type II (PI4KIIα and PI4KIIβ) and type III (PI4KIIIα and PI4KIIIβ) (Li et al., 2021). Distinct structural motifs in each isoform confer unique protein interactions, driving isoform-specific localization and functional roles (Clayton et al., 2013; Boura and Nencka, 2015). See the details of PI4K domain organization in reference (Boura and Nencka, 2015).

The kinase domain of both type II PI4K isoforms contains a cysteine-rich (CCPCC) motif. Palmitoylation of this motif regulates their membrane association and enzymatic activity (Boura and Nencka, 2015). Moreover, type II PI4Ks are insensitive to wortmannin (Guo et al., 2003). PI4KIIα is the most abundant isoform, accounting for half of total PI4P synthesis. It is associated with membranes, primarily the trans-Golgi network (TGN) and late endosomes, which function in cargo sorting during TGN to late endosome trafficking (Wei et al., 2002; Jovi et al., 2012; Boura and Nencka, 2015). PI4KIIβ, meanwhile, is primarily found inactive in the cytosol within heat shock protein 90 (Hsp90) stabilization. Upon palmitoylation, it associates with the membrane of the trafficking vesicles and become active (Balla et al., 2002).

PI4Ks Type III consist of two isoforms, PI4KIIIα (also known as PI4KA) and PI4KIIIβ (also known as PI4KB), both of which are membrane-associated proteins and wortmannin-sensitive. PI4KIIIα mainly localizes in the ER, early cis-Golgi, and plasma membrane (Clayton et al., 2013). PI4KIIIα is recruited to the plasma membrane by association with other regulatory proteins, including TTC7, FAM126, and EFR3B (Nakatsu et al., 2012; Lees et al., 2017). It is responsible for most of the PI4P generation at the plasma membrane. The generation of PI4P at the plasma membrane is important for maintaining lipid composition via the non-vesicular lipid transport. PI4P can be exchanged for lipids being transported, including phosphatidylserine (PS) from the ER to the plasma membrane (Nakatsu et al., 2012), or cholesterol from the ER to the TGN, mediated by the oxysterol-binding protein (OSBP)-related proteins (ORPs; ORP5 and ORP8) (Delfosse et al., 2020; McPhail and Burke, 2023).

PI4KIIIβ is primarily localized at the Golgi, TGN, and Golgi-derived vesicles. Both PI4KIIIβ and PI4Ks type II are mainly responsible for PI4P generation at the Golgi and TGN. This produced PI4P plays a key role in lipid transport via multiple cargoes, including cholesterol, ceramide, and sphingolipid (Weixel et al., 2005; McPhail and Burke, 2023). A small GTPase, Arf1, acts as a recruiter for PI4KIIIβ to localize it to the Golgi for PI4P generation, which is crucial for vesicle formation and transport (Godi et al., 1999; Bilodeau et al., 2020). PI4KIIIβ also associates with the calcium binding protein neuronal calcium sensor-1 (NCS-1) (Taverna et al., 2002) and the Rab11 GTPase (Graaf et al., 2004), which are important in the recruitment of other lipid transport proteins.

#### 2.1.3 PIP4K/PIP5K family

The members of the PIP4K/PIP5K family are classified into three distantly related groups: phosphatidylinositol-4-phosphate-5-kinase type I (PIP5KI), phosphatidylinositol-5-phosphate 4-kinase type II (PIP4K), and phosphatidylinositol-3-phosphate 5-kinase type III (PIKfyve). Both PIP5KI and PIP4K are responsible for the generation of PI(4,5)P2. PIP5KI phosphorylates PI4P at the D5 position, whereas PIP4K phosphorylates PI5P at the D4 position. Since the level of PI4P is much higher than that of PI5P, the major production of PI(4,5)P2 is mediated through PIP5KI activity (van den Bout and Divecha, 2009). PI(4,5)P2 is the most abundant bi-phosphorylated PPIn and is mostly found at the plasma membrane.

PIKfyve is responsible for the generation of PI(3,5)P2 and PI5P. PI(3,5)P2 can be produced by phosphorylation at D5 position of PI3P (McCartney et al., 2014). However, PIKfyve indirectly produces PI5P. It is shown that this enzyme generates a PI(3,5)P2 pool, which is then dephosphorylated by phosphatases, yielding PI5P (Zolov et al., 2012). PIKfyve is mainly involved in the maturation of endosomes from early endosomes to the TGN and lysosome transport (Rivero-Ríos and Weisman, 2022). PI(3,5)P2 is required for the recruitment of the effector protein sorting nexin-1, which participates in late endosome trafficking (Rutherford et al., 2006).

### 2.2 Biosynthesis of inositol phosphates and inositol pyrophosphates

Soluble phosphorylated MI derivatives include inositol phosphates (InsPs), composed of inositol rings bearing one or more phosphate groups at distinct positions. When two phosphates occupy the same position, they form inositol pyrophosphates (PP-InsPs) (Shah et al., 2017). Their biosynthesis pathways via lipid- or glucose-dependent pathways, depending on precursor origin (Figure 1) (Tu-Sekine and Kim, 2022).

In the lipid-dependent pathway, PI(4,5)P2 is converted into 1,2-diacylglycerol (DAG) and Ins(1,4,5)P3 by phospholipase-C (PLC) (Pattni and Banting, 2004). Ins(1,4,5)P3 could be dephosphorylated by inositol polyphosphate-5-phosphatase (INPP5) and inositol polyphosphate-4-phosphatase (INPP4) to Ins(1,4)P2 and Ins(4)P, respectively, for recycling to free MI by the activity of IMPase. Alternatively, Ins(1,4,5)P3 can be further phosphorylated by inositol polyphosphate multikinase (IPMK) into Ins(1,3,4,5)P4, Ins(1,3,4,5,6)P5, and finally by inositol-pentakisphosphate 2-kinase (IPPK) into inositol-6-phosphate (InsP6). Then the activity of inositol hexakisphosphate kinase (IP6K) and diphosphoinositol-pentakisphosphate kinase (PPIP5K) can convert InsP6 to various PP-InsPs (Irvine and Schell, 2001; Tu-Sekine and Kim, 2022; Bizzarri et al., 2023).

The glucose-dependent InsP biosynthesis starts with the conversion of glucose to G6P. Then G6P is converted to inositol-3-phosphate [Ins(3)P] by MIPS activity (Eisenberg, 1967). The phosphate of Ins(3)P can be removed by IMPase to free MI or converted to Ins(3,4)P2 and higher InsP by inositol-tetrakisphosphate 1-kinase (ITPK1) (Raboy and Bowen, 2006). The primary pathway in InsP and PP-InsPs biosynthesis is lipid-dependent, only phosphate starvation stimulates the glucose-dependent pathway (Desfougères et al., 2019).

## 3 Phosphatidylinositol kinases and viral replication

A common strategy for manipulating host cellular biological pathways involves hijacking the function of PIKs, which regulate numerous biological processes. The manipulation of PIKs by representative viruses from each group in the Baltimore classification system is described below and illustrated in Figure 2.

### 3.1 DNA viruses

#### 3.1.1 Group I: double-stranded DNA (dsDNA) viruses

##### 3.1.1.1 Adenoviruses

Adenoviruses are non-enveloped, icosahedral dsDNA viruses measuring 90–100 nm in diameter and containing a genome of approximately 30–37 kbp. They are members of the family *Adenoviridae*. Human adenoviruses belong to the genus *Mastadenovirus* and are usually associated with infections of the respiratory tract, intestinal tract, and eye (Burrell et al., 2017).

The adenovirus E4-ORF1 gene encodes an oncoprotein that promotes viral replication, cell survival, and cellular transformation through activation of PI3K. E4-ORF1 interacts with the regulatory and catalytic subunits of PI3K, elevating their expression levels. PI3K activation requires the formation of an E4-ORF1–PI3K complex in the cytoplasm, which subsequently binds to the membrane-associated cellular protein Discs Large 1 (Dlg1). At the membrane, the resulting complex of three proteins activates PI3K, leading to downstream activation of Akt (Kong et al., 2014). Moreover, PI3K activation upon adenovirus interaction with αv integrins is required for adenovirus internalization. Wortmannin and LY294002, potent PI3K inhibitors, have demonstrated inhibition of adenovirus infection (Li et al., 1998). In addition, LY294002 inhibits activation of the Akt/mTOR pathway and induces early cytopathic effects and caspase-mediated cell death in adenovirus-infected cells (Rajala et al., 2005; Tong et al., 2014).

##### 3.1.1.2 Herpesviruses

Herpesviruses are enveloped dsDNA viruses with highly complex virions and genomes, encoding approximately 70 to 200 proteins. The *Herpesviridae* family is characterized by a dual life cycle, lytic and latent infection, which can establish lifelong persistence host cells. Eight human herpesviruses have been identified: herpes simplex viruses (HSV)-1 and -2, varicella-zoster virus (VZV), cytomegalovirus (CMV), Epstein–Barr virus (EBV), human herpesviruses (HHV)-6 and -7, and Kaposi sarcoma–associated herpesvirus (KSHV, also known as HHV-8).

Human herpesviruses activate PI3K/Akt signaling at multiple stages of the viral life cycle to modulate the cellular environment in favor of viral replication, particularly influencing transcription, translation, cell cycle regulation, suppression of apoptosis, and evasion of the host innate immune response. A comprehensive review is available in reference (Liu and Cohen, 2015). The activation of PI3K/Akt signaling occurs during the entry step, following the binding of HSV-1 to cellular receptors (MacLeod and Minson, 2010). Similarly, this activation is observed upon EBV binding to CD21 on B cells (Barel et al., 2003), and during the interaction of KSHV glycoproteins with integrins (Naranatt et al., 2003). In CMV infection, phosphorylation of the platelet-derived growth factor receptor α (PDGFR-α) triggers its interaction with the p85 subunit of PI3K, leading to Akt activation (Soroceanu et al., 2008). Inhibition of PI3K activity by the PI3K inhibitor LY294002 suppresses HSV-1 entry and fusion, as well as CMV early gene expression and genome replication (Johnson et al., 2001; Tiwari and Shukla, 2010; McFarlane et al., 2011).

To maintain the latent stage of HSV-1, persistent PI3K activation is required and is mediated by nerve growth factor (NGF) binding to the TrkA receptor tyrosine kinase (RTK) (Camarena et al., 2010). Inhibition of PI3K leads to HSV-1 reactivation. Moreover, EBV and KSHV are oncogenic viruses, and PI3K/Akt activation has been observed in malignancies associated with these viruses (Bhatt and Damania, 2012; Chen, 2012). Additionally, aberrant activation of PI3K/Akt signaling is frequently observed in various types of cancer, often resulting from mutations or amplification of genes encoding PI3K catalytic subunits (Bowles et al., 2007). Therefore, targeting the PI3K/Akt signaling pathway represents a critical strategy for drug development aimed at treating both human malignancies and viral infections.

In addition, increased PI4K and PIP5KI activities have been observed in EBV-infected B cells, resulting in elevated levels of PI4P and PI(4,5)P2. These phosphoinositides serve as precursors for the second messengers; 2-diacylglycerol and inositol 1,4,5-trisphosphate, which are required for EBV-induced activation of human B cells (Suzuki et al., 1992).

##### 3.1.1.3 Poxviruses

Poxviruses are large enveloped viruses that belong to the family *Poxviridae* and have brick-shaped or oval structures ranging from 220 to 450 nanometers in length. The *Chordopoxvirinae* subfamily includes several genera, among which the genus *Orthopoxvirus* comprises notable human pathogens such as variola virus (smallpox), vaccinia virus, cowpox virus, and monkeypox virus (MPXV) (Lane and Xiang, 2022).

A previous study identified PI3P and PI4P binding sites on the H7 protein of vaccinia virus, which were found to be essential for viral membrane biogenesis (Kolli et al., 2015). Notably, inhibition of PI4KIIIβ using various bithiazole derivatives significantly reduced MPXV production (Martina et al., 2024). Moreover, PI3K/Akt activation was found to be elevated following infection with poxviruses, including vaccinia, cowpox (Soares et al., 2009), and rabbitpox (myxoma) viruses (Wang G. et al., 2006), contributing to apoptosis suppression (Soares et al., 2009), enhanced viral mRNA translation (Zaborowska and Walsh, 2009), and poxvirus morphogenesis (McNulty et al., 2010). Inhibition of PI3K, either through chemical inhibitors or deletion of its catalytic subunit, resulted in reduced late gene expression and decreased virus production (McNulty et al., 2010; Dunn and Connor, 2012).

#### 3.1.2 Group II: single-stranded DNA (ssDNA) viruses

The viruses in this group have ssDNA genomes and replicate in the nucleus. Examples of viruses include the *Anelloviridae*, *Circoviridae*, and *Parvoviridae* families. Most Group II viruses contain circular genomes, except for parvoviruses. The family *Parvoviridae* consists of two subfamilies: *Parvovirinae*, which includes viruses that infect vertebrates, and *Densovirinae*, which infect insects. The virion is non-enveloped and possesses an icosahedral capsid surrounding its genome. A study using the insect parvovirus Junonia coenia densovirus (JcDV) demonstrated that the PI3K/Akt/TOR pathway is essential during the early stages of infection, likely facilitating the progression of host cells into a phase of the cell cycle suitable for viral replication and non-structural (NS) protein expression. Subsequently, the NS proteins appear to inhibit TOR activity, which suppresses cap-dependent translation, while promoting the preferential translation of viral mRNAs and enhancing viral replication (Salasc et al., 2016).

### 3.2 RNA viruses

#### 3.2.1 Group III: double-stranded RNA (dsRNA) viruses

##### 3.2.1.1 Rotaviruses

Rotaviruses are the most common cause of gastroenteritis in infants and young children. This genus belongs to the *Reoviridae* family. The virion is non-enveloped and contains a triple-layered capsid that encloses a segmented dsRNA genome.

PI3K/Akt/mTOR signaling is essential for the rotavirus life cycle at multiple stages. The binding of the viral capsid to cell surface receptors triggers PI3K activation early in infection, leading to the phosphorylation of Akt and extracellular signal-regulated kinase (ERK), which are crucial for the uncoating process. Subunit E of the V1 domain of V-ATPase directly interacts with phosphorylated PI3K, Akt, and ERK, facilitating proton gradient formation via ATP hydrolysis to acidify the late endosome, thereby enabling virus uncoating (Soliman et al., 2018). Moreover, rotavirus non-structural protein 1 (NSP1) interacts with the PI3K regulatory subunits (p85α and p85β) (Bagchi et al., 2013), leading to the activation of PI3K/Akt signaling. This promotes cell survival or suppresses premature apoptosis, thereby supporting viral production (Bagchi et al., 2010).

In addition, the PI3K inhibitor LY294002 reduces viral production, as evidenced by decreased levels of viral RNA, protein synthesis, and infectious viral particles. These findings confirm that PI3K/Akt/mTOR signaling is essential for sustaining rotavirus infection (Yin et al., 2018).

#### 3.2.2 Group IV: positive-sense single-stranded RNA (+ssRNA) viruses

##### 3.2.2.1 Picornaviruses

Picornaviruses are small, spherical, non-enveloped RNA viruses, and approximately 20–30 nm in diameter. They belong to the *Picornaviridae* family, which comprises five genera: *Enterovirus*, *Rhinovirus*, *Hepatovirus*, *Cardiovirus*, and *Aphthovirus* (Lin et al., 2009). Their genome, approximately 7.5–9 kb in length, is enclosed within an icosahedral capsid (Tapparel et al., 2013).

The replication of most picornaviruses impacts host lipid metabolism. The ROs of picornaviruses initially originate from the ER and TGN, establishing extensive contacts with both the ER and lipid droplets. These contacts are crucial for facilitating the transport of lipids necessary for viral replication (Pattni and Banting, 2004). Increased PI4P levels are also observed upon several picornavirus infections (Belov and van Kuppeveld, 2012).

The viral 3A proteins of Aichivirus (AiV), bovine kobuvirus, poliovirus (PV), coxsackievirus B3 (CVB3), and human rhinovirus 14 (HRV14) are found to associate with PI4KIIIβ (Greninger et al., 2012). The viral 3A protein modulates the recruitment of PI4KIIIβ by activating ADP-ribosylation factor 1 (Arf1) GTPase with guanine nucleotide exchange factor (GBF1). This leads to the accumulation of PI4KIIIβ at the ROs, creating PI4P-enriched membranes (Hsu et al., 2010). The increased PI4P levels at ROs facilitate lipid transport, mediated by lipid transport proteins, enriching sphingolipids and sterols, which are crucial for viral replication (Altan-Bonnet and Balla, 2012). ORPs mediate the transfer of sterol or PS from the ER to other cellular compartments, including viral ROs, by exchanging them for PI4P (Fuggetta et al., 2024). These alterations are critical for several picornaviruses, including PV (Arita, 2014), coxsackievirus (CV) (Dorobantu et al., 2014), rhinovirus (RV) (Spickler et al., 2013), AiV (McPhail et al., 2017), enterovirus 68/71(EV68/71) (van der Schaar et al., 2013), and encephalomyocarditis virus (EMCV) (Dorobantu et al., 2016). In addition, a previous study showed that the recruitment of viral RNA-dependent RNA polymerase (RdRP) of EV71, AiV, and CVB3 to the lipid bilayer of ROs is driven by the overall negative charge of the ROs rather than a specific interaction with PI4P (Dubankova et al., 2017).

Moreover, activation of PI3K/Akt signaling during PV and RV attachment and entry has been observed, resulting in the suppression of apoptosis (Bentley et al., 2007; Autret et al., 2008). A similar mechanism has also been identified in cardioviruses, which suppress apoptosis to maintain infected cell viability. ECMV and coxsackievirus likewise activate PI3K/Akt signaling; however, this activation does not appear to be entry-dependent. Instead, it plays a crucial role in inhibiting apoptosis and promoting viral replication (Esfandiarei et al., 2004; Esfandiarei et al., 2007). An inhibitor of PI4KIII and PI3K, has demonstrated antiviral activity against several picornaviruses (Delang et al., 2012; Siltz et al., 2014; Cheong et al., 2023; Martina et al., 2024).

##### 3.2.2.2 Flaviviruses

Flaviviruses, a genus within the *Flaviviridae* family, are enveloped spherical viruses approximately 50 nm in diameter. The genus includes diverse viruses, such as hepatitis C virus (HCV), dengue virus (DENV), Japanese encephalitis virus (JEV), Zika virus (ZIKV), West Nile virus (WNV), yellow fever virus (YFV), and tick-borne encephalitis virus (TBEV) (van den Elsen et al., 2021).

The involvement of PIKs in viral replication was observed during HCV infection. PI4P, which is primarily localized to the ER membranes, exhibits increased expression and altered redistribution, forming a punctate pattern in the cytoplasm (Deng et al., 2010). It was found that HCV recruited PI4KIIIα to the site of viral replication or the membranous web structure by directly interacting with viral non-structural protein 5A (NS5A) (Bishé et al., 2012). The membranous web is a complex structure of membranes derived from ER membranes, containing double-membrane vesicles (DMVs) induced by HCV (Blanchard and Roingeard, 2018). The interaction of PI4KIIIα and NS5A results in enhanced PI4KIIIα activity and increased PI4P levels (Berger et al., 2011). Impaired interaction between NS5A and PI4KIIIα leads to reduced PI4P levels and alters the morphology of the membranous web, resembling the phenotype observed when PI4KIIIα expression is silenced (Reiss et al., 2013). Additionally, the presence of PI4P at the TGN membrane is essential for HCV secretion (Bishé et al., 2012). PI4KIIIα also regulates the phosphorylation status of NS5A and viral RNA replication (Berger et al., 2011; Reiss et al., 2013). A number of studies have revealed the involvement of PI4KIIIβ as an essential factor for the viral replication of different HCV genotypes (Borawski et al., 2009; Coller et al., 2012; Delang et al., 2012; Zhang et al., 2012). The silencing of PI4KIIIβ results in the inhibition of HCV infection; however, it does not affect HCV membranous web formation (Tai and Salloum, 2011).

The function of PI3Ks is also essential for HCV replication. HCV NS5A can interact with the regulatory subunit p85 of PI3K, thereby freeing the catalytic subunit p110, allowing PI3K to generate PI(3,4)P2 and PI(3,4,5)P3. This leads to Akt recruitment and activation of the PI3K/Akt signaling pathway, which drives cell survival and suppresses apoptosis. The irregular activation of the PI3K/Akt signaling pathway results in the development of hepatocellular carcinoma (Bishé et al., 2012; Cheng et al., 2015). There are a number of PI4KIII inhibitors with antiviral activity against HCV genotype 1b, including PIK-93, Enviroxime, and AL-9, which have shown inhibitory effects on both PI4KIIIα and PI4KIIIβ (Borawski et al., 2009; Delang et al., 2012). PIK-93 and AL-9 also inhibit certain types of PI3Ks.

Moreover, PI4KIIIβ was identified as an essential factor in ZIKV replication. PI4P is enriched at ZIKV ROs, and treatment with the PI4KIIIβ inhibitor, bithiazole, was found to inhibit ZIKV replication (Martina et al., 2021). Particularly, enriched PI4P contributes a negative charge to the lipid bilayer and mediates electrostatic interaction between NS1 and the ER membrane (Ci et al., 2021). The overexpression of a lipid phosphatase, Sac1, which dephosphorylates PI4P, disrupted NS1-induced ER membrane remodeling and impaired ZIKV replication. The electrostatic interaction between NS1 of flaviviruses is crucial for the induction of ROs by binding to negatively charged lipids and might also be applicable to other flaviviruses (Martina et al., 2021). ZIKV and other flaviviruses, such as DENV and West Nile virus (WNV), form convoluted membranes and vesicle packets within the ER as their ROs, while HCV forms double-membrane vesicles (Mazeaud et al., 2021).

However, the replication of DENV is independent of both PI4KIIIα and PI4KIIIβ (Heaton et al., 2010; Delang et al., 2012). Silencing of PI4KIIIα had no effects on DENV replication (Reiss et al., 2011). WNV infection is also independent of PI4P, as no alterations in PI4P distribution and colocalization with viral double-stranded RNA (dsRNA) have been observed. The treatment with PIK-93, a PI4K inhibitor, had no effect on WNV replication (Martín-Acebes et al., 2011). Nevertheless, both DENV and WNV manipulate host lipid metabolism. Fatty acid synthase (FASN) is recruited to the replication sites of both DENV and WNV, and treatment with FASN inhibitors such as Cerulenin and C75 significantly inhibited viral replication (Mackenzie et al., 2007; Heaton et al., 2010). Moreover, itraconazole and posaconazole have been shown to inhibit DENV and ZIKV by targeting oxysterol-binding protein (OSBP) function in the redistribution of cholesterol (Meutiawati et al., 2018).

Flaviviruses usually activate apoptosis in the late stage of infection; however, they also initiate survival signaling to create and prolong a favorable cellular environment for their replication through PI3K/Akt/mTOR pathway. Upon interaction with DENV serotype 2 (DENV-2) and Japanese encephalitis virus (JEV), apoptosis is inhibited by activating the PI3K/Akt pathway at an early stage of viral infection (Lee et al., 2005). However, PI3K/Akt signaling is not required for JEV and DENV-2 replication, as LY294002, a PI3K inhibitor, has no effects on viral RNA replication, viral protein expression, and virion production (Lee et al., 2005). Furthermore, PI3K signaling regulates the type I IFN (IFN-I) response, which is important for controlling WNV infection. The inhibition of PI3Ks by 3-methyl adenine (3-MA), Wortmannin, and LY294002 increased viral production (Wang et al., 2017).

##### 3.2.2.3 Coronaviruses

Coronaviruses are members of the family *Coronaviridae* and are characterized as large, spherical, enveloped viruses that feature prominent spike (S) glycoproteins protruding from their envelope. They have an approximate diameter of 118–140 nm. Their genome consists of +ssRNA, ranging from 25 to 32 kb and containing 7–10 open reading frames (ORFs) (Chen et al., 2020). Three coronaviruses have caused severe disease in humans: severe acute respiratory syndrome coronavirus 1 (SARS-CoV-1), Middle East respiratory syndrome coronavirus (MERS-CoV), and SARS-CoV-2.

PI4KIIIβ was identified as being involved in the entry of SARS-CoV-1, mediated by angiotensin I-converting enzyme 2 (ACE2) receptors (Yang et al., 2012). Silencing of PI4KIIIβ resulted in the inhibition of SARS-CoV-1 entry. PI4KIIIβ is important for regulating lipid membrane composition; an increase in PI4P levels in the organelle membrane is also favorable for SARS-CoV-1 (Yang et al., 2012). Moreover, PIKfyve, the lipid kinase responsible for PI(3,5)P2 production, which regulates early endosome to late endosome maturation, is crucial for SARS-CoV-2, MERS-CoV, and murine hepatitis virus (MHV) entry. Inhibition of PIKfyve by apilimod significantly reduces viral entry (Ou et al., 2020).

Furthermore, PI3K also participates in MERS-CoV replication by regulating cell proliferation and apoptosis through the PI3K/Akt/mTOR signaling pathways. Wortmannin, a PI3K inhibitor, also inhibited MERS-CoV infection (Kindrachuk et al., 2015).

#### 3.2.3 Group V: negative-sense single-stranded RNA (−ssRNA) viruses

##### 3.2.3.1 Ebolaviruses

The *Ebolavirus* genus belongs to the *Filoviridae* family of viruses. It includes five species: Bundibugyo ebolavirus (BDBV), Zaire ebolavirus (ZEBOV), Reston ebolavirus (RESTV), Sudan ebolavirus (SUDV), and Taï Forest ebolavirus (TAFV) (Jain et al., 2021).

During viral entry, Ebolaviruses (EBOV) rely on the interaction between its viral glycoprotein and the host cellular protein Niemann-Pick C1 (NPC1), which is located in late endosomes and lysosomes. PIKfyve, which is responsible for PI(3,5)P2 production to regulate endosome maturation, thus mediates the transport of EBOV to NPC1-positive late endosomes (Qiu et al., 2018). The inhibition of PIKfyve using apilimod was also shown to inhibit the entry of ZEBOV (Kang et al., 2020).

Moreover, the interaction between viral VP40 and the plasma membrane is critical for the assembly and budding of EBOV. This step requires high concentrations of PI(4,5)P2, PI3P, and PI(3,4,5)P3. Especially, the enrichment of PI(4,5)P2 at the plasma membrane, which is mainly generated by PIP5KI, changes the lipid composition and induces membrane curvature during assembly and budding (Gc et al., 2016; Johnson et al., 2016).

Furthermore, it has been identified that PI3KC1 and Akt activation participates in the regulation of ZEBOV entry through modulation of Rac1, which is the regulatory protein involved in endocytosis. Inhibition of PI3K or Akt activation resulted in reduced viral entry (Saeed et al., 2008).

##### 3.2.3.2 Orthomyxoviruses

Viruses of the *Orthomyxoviridae* family are characterized by segmented −ssRNA genomes and surface glycoproteins hemagglutinin (HA) and neuraminidase (NA), which are crucial for viral entry, subtype distinction, and infectivity. Their genomes typically consist of 6 to 8 RNA segments, each packaged into ribonucleoprotein (RNP) complexes that encode essential proteins for replication (Foster et al., 2018). Influenza A, B, and C viruses are the most notable orthomyxoviruses, responsible for seasonal influenza and periodic pandemics in both humans and animals.

Activation of PI3K/Akt signaling by influenza A virus (IAV) occurs at multiple stages of viral replication and can have either pro-viral or anti-viral effects. Binding of IAV to sialic acids on the host cell surface, followed by endocytosis, requires activation of PI3K/Akt signaling to facilitate viral internalization (Ehrhardt and Ludwig, 2009). PI3K/Akt signaling activation is initiated through clustering of RTKs, such as the epidermal growth factor receptor (EGFR) (Eierhoff et al., 2010). However, PI3K signaling triggered by pathogen recognition receptors also lead to host innate immune responses activation (Ayllon et al., 2012). Moreover, IAV directly activates PI3K/Akt signaling through interaction between its N1 protein and the p85 regulatory subunit of PI3KCI, ultimately leading to inhibition of apoptosis (Hale et al., 2006; Shin et al., 2007). Although only early PI3K activation was observed with influenza B virus infection (Ehrhardt et al., 2007).

During viral assembly, the viral ribonucleoprotein (vRNP) of IAV must be transported from the nucleus to the plasma membrane. IAV has been shown to induce cellular PI4P levels and alter its localization via ATG16L1, which is essential for autophagosome formation, thereby promoting vRNP trafficking (Alemany et al., 2025).

#### 3.2.4 Group VI: single-stranded RNA-reverse transcription (RT) viruses

##### 3.2.4.1 Retroviruses

These viruses belong to the family *Retroviridae*. The virions of retroviruses contain reverse transcriptase, which converts their RNA genome into DNA that subsequently integrates into the host genome. Examples of retroviruses include the human immunodeficiency virus (HIV), which causes acquired immunodeficiency syndrome (AIDS), the human T-lymphotropic virus (HTLV), which is associated with certain types of leukemia and lymphoma, and murine leukemia virus (MLV) (Chameettachal et al., 2023).

PtdIns kinases are identified to be involved in the human immunodeficiency virus-1 (HIV-1) replication cycle. The interaction of HIV-1 with the cell surface receptor CD4 can activate PI3K/Akt signaling pathways, which are important in HIV-1 entry, increasing cell survival and viral spread, and interfering with the immune response (Hamada et al., 2019; Pasquereau and Herbein, 2022). PI3KCI, α isoform (with the p110α catalytic subunit), which is responsible for PI(3,4,5)P3 generation, is identified as a crucial factor for HIV-1 entry and fusion. PIK-75, a PI3K p110α isoform-specific inhibitor, can inhibit HIV-1 entry (Hamada et al., 2019). Additionally, the negative factor (Nef) of HIV-1 interacts with p85, a regulatory subunit of PI3K, which is required to activate Nef-associated p21-activated kinase (PAK) (Wolf et al., 2001). The activation of PAK suppresses apoptosis and T cell development, leading to facilitated viral replication (Wolf et al., 2001). The inhibition of PI3K showed reduced HIV-1 production (Linnemann et al., 2002).

PIP5KI, which is responsible for PI(4,5)P2 production, is also involved in HIV-1 entry and assembly. PI(4,5)P2 is required for actin cytoskeleton remodeling, regulating endocytosis and thereby viral entry (Ling et al., 2006; Gonzales et al., 2020). PI(4,5)P2 also mediates the binding of Gag polyprotein precursors (Pr55Gag) to the plasma membrane in viral assembly. Particularly, the downregulation of PIP5K1α and PIP5K1γ isoforms impairs the targeting of Pr55Gag to the plasma membrane (Gonzales et al., 2020).

#### 3.2.5 Group VII: dsDNA-RT viruses

These viruses possess a dsDNA genome and utilize reverse transcriptase to replicate their genome from transcribed RNA. An example is the *Hepadnaviridae* family.

##### 3.2.5.1 Hepadnaviruses

These enveloped viruses are characterized by a partially dsDNA genome and preferentially infects and replicates within liver cells. They are associated with both acute and chronic hepatitis, which can progress to cirrhosis and liver cancer. The most well-known member of this group is hepatitis B virus (HBV), a major human pathogen.

Activation of PI3K/Akt/mTOR signaling is observed during HBV entry. However, transient treatment with the PI3K inhibitor LY294002 has no effect on the entry process, suggesting that HBV-induced Akt activation is not essential for viral entry. Notably, prolonged treatment with PI3K/Akt/mTOR inhibitors, including LY294002, an Akt inhibitor, and rapamycin, results in increased levels of HBV capsids and capsid DNA, thereby enhancing viral replication (Xiang and Wang, 2018).

HBx, a protein encoded by HBV, stimulates viral replication and contributes to the development of HBV-associated hepatocellular carcinoma (HCC). HBx has been found to activate PI3K/Akt signaling; however, this activation leads to a reduction in HBV replication. Although Akt activation by HBx appears to negatively regulate HBV replication, it is also essential for the suppression of apoptosis, which may support persistent, non-cytopathic HBV replication. Akt modulates HBV replication by decreasing the activity of the transcription factor hepatocyte nuclear factor 4α (HNF4α). These findings highlight the crucial role of HBx in balancing HBV replication and host cell survival through PI3K/Akt signaling (Rawat and Bouchard, 2015). Additionally, Akt1 activation is among the most consistent features observed in HBV-induced HCC (Boyault et al., 2007).

## 4 Phosphatidylinositol kinases as an antiviral target

The biosynthesis of PtdIns and its phosphorylated derivatives involves a range of cellular biological processes. As obligate parasites, viruses rely on host cellular machinery to support their replication. A common strategy employed by viruses to manipulate these processes and create a favorable environment for their replication involves regulating the functions of PIKs and the expression levels of PPIns (Figure 2). Modulating these PIKs is crucial for viral entry, fusion, genome replication, translation, assembly, and release across multiple viral families (Buchkovich et al., 2008; Dunn and Connor, 2012), highlighting PIKs as promising antiviral targets.

Several pan- or isoform-specific PI3K, PI4K, and PIKfyve inhibitors, include LY294002, PIK-75, PIK-93, enviroxime, bithiazole derivatives, CUR-N399, and apilimod, have demonstrated antiviral activity against multiple viruses (Table 1 and Figure 2) (Walker et al., 2000; Delang et al., 2012; Martina et al., 2021; Li et al., 2024).

**TABLE 1:** Inhibitors of phosphatidylinositol kinases with board-spectrum antiviral activity.

Inhibitors	Targeted PIKs	Virus group and species or strain	Findings	References
LY294002	PI3Kα, PI3Kδ, PI3Kβ	HAdV-2	Inhibits viral entry, conc. 100 μM (SW480 cells)	Li et al., 1998
		HAdV-19	Inhibits Akt activation, conc. 20 μM (HCF cells)	Rajala et al., 2005
		HSV-1	Inhibits viral entry, conc. 0.05–0.5 mM (RPE, HeLa and CF cells)	Tiwari and Shukla, 2010
		HCMV	Inhibits virus replication, conc. 10 μM (HFFF2 cells)	McFarlane et al., 2011
			conc. 1–20 μM (HEL fibroblasts)	Johnson et al., 2001
		CyHV-2	Inhibits virus replication and protein expression (GiCF cells)	Song et al., 2024
		RVA-SA11	Inhibits virus replication, conc. 1, 5 μM (Caco2 cells)	Yin et al., 2018
		CVB	Promotes CVB3-induced CPE and apoptosis	Chen et al., 2014
		IAV A/WSN/33 (H1N1)	Reduces virus replication, attenuates lung injury in mice	Tang et al., 2023
		HIV-1	Inhibits viral entry, conc. 3–10 μM (TZM-bl cells)	Hamada et al., 2019
PIK-75	PI3Kα	IAV	Inhibits virus replication in A549 cells	Perwitasari et al., 2015
		A/Anhui/1/2013 (H7N9)	IC_50_: 0.04 μM	
		A/California/04/09 (pdmH1N1)	IC_50_: 0.32 μM	
		A/Philippines/2/82-X79 (H3N2)	IC_50_: 0.40 μM	
		HIV-1	Inhibits viral entry, conc. 3–30 nM (TZM-bl cells)	Hamada et al., 2019
PIK-93	PI4KIIIβ, PI3Kγ, PI3Kα	AiV	inhibits virus replication, EC_50_: 0.60 μM (HeLa cells)	Greninger et al., 2012
		CVB3	Inhibits virus replication, conc. 1 μM (BGM kidney cells)	Schaar et al., 2012
		EV71	Inhibits virus replication, conc. 0.25 μM (RD cells)	Xiao et al., 2017
		HRV	Inhibits virus replication, (Cells) mean EC_50_ ± SD	Mello et al., 2014
		HRV-C15	(HeLa) 285 ± 258 nM (HAE) 225 ± 103 nM	
		HRV-C11	(HeLa cells) 90 ± 10 nM (HAE) 342 ± 81 nM	
		HRV-C25	(HeLa) 57 ± 33 nM	
		HRV-C24	(HeLa) 75 ± 9 nM	
		HRV-A16	(HeLa) 574 ± 115 nM (HAE) 127 ± 50 nM	
		PV	Inhibits virus replication Mean EC_50_ ± SD: 0.14 ± 0.0086 μM (RD cells)	Arita et al., 2011
		HCV	Inhibits virus replication (Huh-7.5) mean IC_50_ ± SD (Huh-7) mean EC_50_ ± SD	Borawski et al., 2009; Delang et al., 2012; Delang et al., 2018
		Genotype 1a	(Huh-7.5) 0.098 ± 0.05 μM (Huh-7) 0.47 ± 0.1 μM	
		Genotype 1b	(Huh-7.5) 0.05 ± 0.01 μM	
		Genotype 1b (Huh 5–2)	(Huh-7) 0.28 ± 0.07 μM	
		Genotype 1b (Huh 9–13)	(Huh-7) 0.17 ± 0.1 μM	
		Genotype 2a	(Huh-7.5) 0.39 ± 0.04 μM (Huh-7) 5.8 ± 0.5 μM	
		Genotype 4a	(Huh-7) 0.72 ± 0.01 μM	
		SARS-CoV-2	Inhibits viral entry, conc. 0.1–10 μmol/L (293T-ACE2 stable cell lines)	Moore et al., 2022
Enviroxime (LY122772)	PI4KIIIβ, PI4KIIIα	MPXV	(VeroE6) EC_50_: 4.75 μM	Martina et al., 2024
		CVB1	Combination of 50 mg/kg enviroxime and 3.125–6.25 mg/kg disoxaril synergistically inhibits virus replication in mice	Nikolaeva-Glomb and Galabov, 2004
		CVB3	Inhibits virus replication, EC_50_: 0.7 μM	Martina et al., 2024
		EV68	(HeLa) EC_50_: 0.154 μM
		EV71	EC_50_: 0.0303 μM
		HRV14	EC_50_: 0.11 μM
		HRV16	(HeLa) EC_50_: 0.042 μM
		HRV54	EC_50_: 0.120 μM
		PV1	EC_50_: 0.19 μM
		PV	(L2OB and RD) MIC of 0.06 μg/ml	Al-khayat and Ahmad, 2012
		Rubella virus	(HeLa and WISH) MIC of 0.125 μg/ml	
		HCV	Inhibits virus replication (Huh-7) mean EC_50_ ± SD or only EC_50_ indicated	Delang et al., 2012; Delang et al., 2018
		Genotype 1a	0.49 ± 0.07 μM	
		Genotype 1b	0.22 μM	
		Genotype 1b (Huh 5–2)	0.33 ± 0.1 μM	
		Genotype 1b (Huh 9–13)	0.22 ± 0.06 μM	
		Genotype 2a	2.3 ± 0.8 μM	
		Genotype 4a	0.20 ± 0.1 μM	
		HCoV-229E	(Huh7) EC_50_: 4.75 μM	Martina et al., 2024
		SARS-CoV-2	(VeroE6) EC_50_: 0.57 μM	
Bithiazole derivatives	PI4KIIIβ	Different substituents on bithiazole derivatives showed varying antiviral activity across various viruses and cell types. (Cells) Ranges of EC_50_ or IC_50_ values		(Martina et al., 2021; Martina et al., 2024)
		MPXV	(VeroE6) EC_50_: 3–11 μM	
		EV68	(HeLa) EC_50_: 0.41–3.22 μM	
		EV71	(VeroE6) EC_50_: 0.05–0.03 μM	
		HRV2	(HeLa) IC_50_: 0.39–9.70 μM	
		HRV14	(HeLa) IC_50_: 0.48–15.30 μM	
		HRV16	(HeLa) EC_50_: 0.145–1.6 μM	
		YFV	(VeroE6) EC_50_: 1.05–1.52 μM	
		ZIKV	(VeroE6) EC_50_: 1.88–4.59 μM (Huh-7) EC_50_: 1.64–6.51 μM (Huh-7) IC_50_: 0.51–13.79 μM	
		HCoV-229E	(Huh-7) EC_50_: 0.55–0.94 μM	
		SARS-CoV-2	(VeroE6) EC_50_: 0.57–9.67 μM (Calu3) EC_50_: 2.71–11.2 μM (Calu3) IC_50_: 1.57–7.45 μM	
CUR-N399	PI4KIIIβ	Enterovirus A, B, C, D	EC_50_: 2.5–53 nM	Cheong et al., 2023
		Human rhinovirus A, B	EC_50_: 2.8–53 nM	
Apilimod	PIKfyve	HRV14	Inhibits virus replication (HeLa) IC_50_: 12.3 μM	Baker et al., 2024
		HRV1B	(HeLa) IC_50_: 0.52 μM	
		HCoV-229E	IC_50_: 0.04 μM	
		HCoV-OC43	IC_50_: 0.007 μM	
		MERS-CoV	Reduces viral entry, conc. 10–1,000 nM (HeLa/hDPP4 cells)	Ou et al., 2020
		SARS-CoV-2	(VeroE6) EC_50_ < 6.9 nM	Su et al., 2022
			(VeroE6) IC_50_ ∼10 nM	Kang et al., 2020
			Reduces viral entry, conc. 10–1,000 nM (293/hACE2 cells)	Ou et al., 2020
		MHV	Reduces viral entry, conc. 10–1,000 nM (HeLa/mCEACAM cells)	
		ZEBOV	Reduces viral entry, IC_50_ ∼50 nM (MA104 cells)	Kang et al., 2020
		Various IAV strains	(MDCK) IC_50_: 3.8–24.6 μM	Baker et al., 2024
		IAV PR8 A/Puerto Rico/8/1934(H1N1)	Inhibits body weight loss in BALB/c mice Dose: 2 mg/mL daily	
		IBV Florida/4/2006	(MDCK) IC_50_: 16.4 μM	
		RSV A2	Inhibits body weight loss in BALB/c mice Dose: 2 mg/mL daily (HEp-2) IC_50_: 19.6 μM	
		PIV3 C243 strain	(LLC-MK2 7.1) IC_50_: 31.1 μM	

AiV, Aichivirus; BGM, buffalo green monkey; CPE, cytopathic effect; CVA21, coxsackievirus A21; CVB, coxsackievirus B; CVB3, coxsackievirus B3; CyHV-2, cyprinid herpesvirus 2; EV68, enterovirus 68; EV71, enterovirus 71; HAdV-2, human adenovirus 2; HAdV-19, human adenovirus 19; HCMV, human cytomegalovirus; HCoV-229E, human coronavirus 229E; HCoV-OC43, human coronavirus OC43; HCV, hepatitis C virus; HIV-1, human Immunodeficiency virus type 1; HSV-1, herpes simplex virus type 1; HRV, human rhinoviruses; HRV14, human rhinovirus 14; HRV-A16, human rhinovirus A16; HRV-C, human rhinovirus C; IAV, influenza A virus; IBV, influenza B virus; MIC, minimal inhibitory concentration; MHV, mouse hepatitis virus; MPXV, monkeypox virus; PIV3, human parainfluenza virus type 3; PV, poliovirus; PV1, poliovirus type 1; RVA-SA11, simian rotavirus SA11; RSV A2, respiratory syncytial virus A2; ZEBOV, Zaire ebolavirus.

LY294002 is a PI3K inhibitor that has demonstrated potent anti-tumor activity (Hu et al., 2000; Bar et al., 2005; Chang et al., 2015; Abdallah et al., 2020). However, in cancer study, it exhibited unfavorable pharmacological properties, such as limited solubility, short half-life, and off-target activities, that impede the achievement of therapeutic levels and may result in undesirable clinical effects (Gupta et al., 2003). Additionally, SF1126, a prodrug consisting of LY294002 conjugated to an RGDS (Arg-Gly-Asp-Ser) peptide, has demonstrated favorable pharmacokinetics and good tolerability in animal models (Garlich et al., 2008). Moreover, phase I clinical trials of SF1126 in patients with malignancies confirmed its tolerability and demonstrated efficacy across multiple types of human cancers (Chiorean et al., 2009; Mahadevan et al., 2012).

A study in mice demonstrated that PIK-75, a PI3K inhibitor, effectively suppressed tumor cell growth (Huang et al., 2022). However, it also targets other kinases, including DNA-dependent protein kinase (DNA-PK), raising concerns about off-target effects and poor solubility, which hinder the achievement of therapeutic concentrations (Jamieson et al., 2011; Talekar et al., 2012). Notably, the maximum serum concentration (C_*max*_) reached approximately 8 μM in mice, exceeding the IC_50_ values observed in *in vitro* antiviral assays against IAV and HIV-1 (Table 1), suggesting potential use in antiviral treatment. Furthermore, the development of a PIK-75 nanosuspension improved solubility and enhanced activity in both *in vitro* assay and mouse models (Talekar et al., 2013).

PIK-93 primarily inhibits PI4KIIIβ and also targets PI3Kγ and PI3Kα. In a mouse model, combination treatment with PIK-93 and a monoclonal antibody suppressed tumor growth and enhanced immune cell activity, thereby improving the efficacy of cancer immunotherapy (Lin et al., 2023). The IC_50_ or EC_50_ values of PIK-93 in *in vitro* antiviral assays against several viruses (Table 1) were below 1 μM, suggesting potential as an antiviral agent; however, data on its C_*max*_ are still lacking.

Enviroxime is an antiviral agent that targets the viral 3A protein of rhinoviruses and enteroviruses, thereby blocking viral replication (Heinz and Vance, 1995). It primarily inhibits PI4KIIIβ and exhibits some activity against PI4KIIIα. Enviroxime has also shown potential inhibition of various genotypes of HCV, coronaviruses, rubella virus, and MPXV. Despite its potent antiviral activity in *in vitro* assays, clinical trials revealed unfavorable pharmacokinetics, undesirable side effects, and limited efficacy (Miller et al., 1985; Garrido et al., 2021). Plasma levels of enviroxime were notably low, with concentrations around 4 ng/ml (approximately 0.01 μM) (Bopp and Miner, 1982), substantially below the antiviral IC_50_ values (Table 1). However, co-administration of enviroxime with disoxaril synergistically inhibited coxsackievirus B1 (CVB1) replication in mice (Nikolaeva-Glomb and Galabov, 2004).

Bithiazole, a compound composed of two linked thiazole rings, primarily inhibits PI4KIIIβ. Derivatives bearing aliphatic or polar functional groups on the right side of the bithiazole scaffold exhibit broad-spectrum antiviral activity against various viral families, with IC_50_ values in the low micromolar range (Table 1). Furthermore, antiviral assays using a human-derived respiratory tissue model (MucilAir) demonstrated effective inhibition of human rhinovirus A16 (HRV-A16), suggesting potential for treating respiratory viral infections (Martina et al., 2024).

CUR-N399 is a PI4KIIIβ inhibitor that exhibits potent broad-spectrum antiviral activity against various genera of picornaviruses in low nanomolar concentrations, displays mild toxicity, and confers protection against lethal EV71 infection in mice (Cheong et al., 2023; Cheong et al., 2023), suggesting potential utility in antiviral therapy. Notably, a phase I clinical trial of CUR-N399 has been conducted to evaluate its safety, tolerability, and pharmacokinetic profile in healthy adults (NCT05016687).

Although apilimod demonstrated potent *in vitro* antiviral activity against various viral families, its poor pharmacokinetics, characterized by low plasma concentration and poor bioavailability, led to inefficacy during clinical trials (Sands et al., 2010; Krausz et al., 2012). A single oral dose of apilimod dimesylate at 15 mg yielded a C_*max*_ of approximately 225 ng/mL (0.368 μM) at 1 h; however, by 6 h, the concentration dropped below 50 ng/mL (0.008 μM) (Ikonomov et al., 2019), which is lower than the desired effective concentration required for viral inhibition (Table 1).

PIK inhibitors, such as PIK-75, PIK-93, bithiazole derivatives, and CUR-N399, demonstrate antiviral activity against various viruses and possess favorable pharmacological properties, highlighting their potential as candidates for antiviral therapy. However, their inhibitory effects remain limited to specific viral families or select members within the same genus. Most reported activity targets picornaviruses; certain flaviviruses; coronaviruses; influenza viruses; other RNA viruses, such as ZEBOV, rubella virus, human parainfluenza virus, and respiratory syncytial virus (RSV); retroviruses such as HIV-1; and DNA viruses including HSV, HCMV, and MPXV.

## 5 Inositol monophosphatase as a broad-spectrum antiviral target

Although viruses within the same genus may share similarities, their replication depends on distinct PIK subtypes. For instance, DENV and WNV, both flaviviruses, do not require PI4K activity, unlike HCV. Interestingly, PI3K inhibition has been shown to enhance WNV production by suppressing PI3K signaling, which in turn impairs the IFN-I response (Wang et al., 2017). Additionally, the PI3K inhibitor LY294002 increases HBV replication (Xiang and Wang, 2018). Notably, viral genotype influences PIK dependency: different HCV genotypes exhibit differing reliance on PI4KIIIα or PI4KIIIβ isoforms, resulting in variable sensitivity to their corresponding PI4KIII inhibitors (Delang et al., 2012).

Our previous study on the elucidation of the antiviral mechanisms of ivermectin (IVM), an antiparasitic agent with potent broad-spectrum antiviral activity, demonstrated the inhibition of IMPase as one of its antiviral mechanisms (Jitobaom et al., 2024). IMPase generates free MI for both *de novo* inositol biosynthesis from glucose and the recycling of PPIns and InsPs. IVM binds to IMPase and inhibits its activity, resulting in the overall reduction of cellular myo-inositol levels, and inhibits virus replication of DENV-2, ZIKV, and SARS-CoV-2 (Jitobaom et al., 2024), which modulate PIKs differently. The inhibition of IMPase activity might provide a broader antiviral approach.

## 6 Future outlook

Compare to the use of PIK inhibitors, which may face an issue with the complexity of PIK subtypes, targeting IMPase may provide a benefit of broader coverage, as it affects all types of PtdIns. This may naturally result in broader effects on multiple cellular processes and increase the risk of side effects. Interfering with cellular machinery always carries the risk of detrimental effects on essential cellular functions, which may lead to adverse outcomes. However, our recent finding that ivermectin can inhibit IMPase, along with the well-established mechanism of lithium involving inhibition of IMPase, suggest that inhibiting this enzyme does not necessarily result in serious adverse effects (Jitobaom et al., 2024). Both ivermectin and lithium have been extensively used. While ivermectin is considered to have a good safety profile (Canga et al., 2008), lithium is characterized by a narrow therapeutic window (Gitlin, 2016). This difference suggests that the higher toxicity of lithium may involve additional mechanisms. Both ivermectin and lithium may act through multiple mechanisms, and it remains to be determined whether novel IMPase inhibitors with higher specificity might result in reduced adverse effects and improved safety profiles.

Another aspect of non-antiviral effects of IMPase inhibitors is that some may be beneficial. Both ivermectin and lithium exhibit anti-inflammatory activity (Seth et al., 2016; Murru et al., 2020; Qaswal et al., 2021; Zaidi and Dehgani-Mobaraki, 2022). This is because PtdIns, PPIns, and InsP are involved in the inflammatory signaling pathways. An anti-inflammatory effect may be beneficial in the treatment of viral diseases, as it helps mitigate tissue damage, illustrated by the use of steroid in COVID-19 therapy (Bahsoun et al., 2023).

Host-targeting and drug repurposing for antiviral development are an area intensely pursued by many research groups, particularly during the COVID-19 pandemic. Several drugs were evaluated in clinical trials but were found to be ineffective. Multiple factors contributed to these failures, including differences in SARS-CoV-2 cell entry mechanisms between cell lines used for antiviral testing and human lung tissue, which likely explained the lack of clinical efficacy observed with chloroquine (Das et al., 2021). In many cases, *in vitro* IC_50_ values exceed the concentrations achievable *in vivo*. The fact that these repurposed drugs were not specifically developed for this indication contributes to their low potency and the lack of clinical efficacy.

Identification of antiviral mechanisms can provide therapeutic targets for the development of novel drugs with enhanced potency and clinical efficacy. Structure-based drug design, coupled with advancement in artificial intelligence, can accelerate drug discovery by leveraging knowledge of specific target enzymes. Effective broad-spectrum antivirals are critical for mitigating and controlling pandemics. Strengthening efforts in this area should be considered an important component of the preparedness for future pandemics.
